# Identification of valid reference genes for mRNA and microRNA normalisation in prostate cancer cell lines

**DOI:** 10.1038/s41598-018-19458-z

**Published:** 2018-01-31

**Authors:** Hui Zhao, Teng-Fei Ma, Jie Lin, Lin-Lin Liu, Wei-Jie Sun, Li-Xia Guo, Si-Qi Wang, Newton O. Otecko, Ya-Ping Zhang

**Affiliations:** 1 State Key Laboratory for Conservation and Utilization of Bio-resource, Yunnan University, Kunming, China; 2 Key Laboratory for Animal Genetic Diversity and Evolution of High Education in Yunnan Province, Yunnan University, Kunming, Yunnan China; 3 School of Life Science, Yunnan University, Kunming, 650091 China; 4 State Key Laboratory of Genetic Resources and Evolution & Yunnan Laboratory of Molecular Biology of Domestic Animals & Germplasm Bank of Wild Species, Kunming Institute of Zoology, Chinese Academy of Sciences, Kunming, 650223 China; 5 Kunming College of Life Science, University of Chinese Academy of Sciences, Kunming, 650204 China

## Abstract

RT-qPCR offers high sensitivity, for accurate interpretations of qPCR results however, normalisation using suitable reference genes is fundamental. Androgens can regulate transcriptional expression including reference gene expression in prostate cancer. In this study, we evaluated ten mRNA and six non-protein coding RNA reference genes in five prostate cell lines under varied dihydrotestosterone (DHT) treatments. We validated the effects of DHT-treatments using media containing charcoal-stripped serum prior to DHT stimulation on the test samples by Western blot experiments. Reference gene expression stability was analysed using three programs (geNorm, NormFinder and BestKeeper), and the recommended comprehensive ranking is provided. Our results reveal that *ACTB* and *GAPDH*, and *miR-16* and *miR-1228-3p* are the most suitable mRNA and miRNA reference genes across all cell lines, respectively. Considering prostate cancer cell types, *ACTB/GAPDH* and *ACTB/HPRT1* are the most suitable reference gene combinations for mRNA analysis, and *miR-16/miR-1228-3p* and *RNU6-2/RNU43* for miRNA analysis in AR+, and AR− and normal cell lines, respectively. Comparison of relative target gene (*PCA3* and *miR-141*) expression reveals different patterns depending on reference genes used for normalisation. To our knowledge, this is the first report on validation of reference genes under different DHT treatments in prostate cancer cells. This study provides insights for discovery of reliable DHT-regulated genes in prostate cells.

## Introduction

Prostate cancer is a common malignancy and the second most prominent cause of cancer-related deaths in Western countries^1^. Prostate cancer prognosis depends on androgens, which affect gene expression via the androgen receptor (AR)^2^. Androgen-deprivation therapy is the first-line treatment for prostate cancer, but fatal castration-resistant prostate cancer (CRPC) often develops, also called androgen-independent prostate cancer. Indeed, several studies show that CRPC may be androgen-dependent in cancer cells themselves, producing androgens during androgen ablation^3–6^. Therefore, identification of androgen-regulated genes as therapeutic targets in prostate cancer is a significant step toward discovering mechanisms of prostate cancer development and progression.

Several methods including reverse transcription quantitative polymerase chain reaction (RT-qPCR), and high throughput methods (microarrays and next generation sequencing) have been developed to measure relative gene expression. RT-qPCR is a powerful technique because of its high sensitivity, relatively low cost, and high time efficiency. When expression is quantified through high throughput methods, verification of interesting expression differences is commonly implemented using RT-qPCR^7^. To avoid bias during qPCR analysis, normalisation of gene expression data using reference genes across the samples under study is an essential step. Thus, qPCR accuracy relies on the expression stability of the internal reference gene, which should be sufficiently abundant and stably expressed among various tissues and cell lines under variable experimental conditions. However, a universal reference gene for all samples does not exist in practice^8^, and biased normalisation can cause inaccurate quantification and incorrect conclusions^8–10^. Therefore, to obtain biologically meaningful expression data, it is fundamental to validate suitable reference genes under different conditions.

Reference genes such as *ACTB*, *ALAS1*, *GAPDH*, *HPRT1*, *K-ALPHA-1*, *RPL13A*, *SDHA*, and *TBP* have been validated to be optimal for normalisation in prostate cancer tumor and normal tissues^11–14^, as well as in primary culture of prostate cancer cells^15^. Also, microRNAs (miRNAs) have gained attention in cancer research due to their role in oncogenesis and tumor metastasis^16^. Some studies suggest that suitable reference genes can be identified by miRNA expression assessments in prostate cancer tissues^17,18^ and in circulating miRNA in prostate cancer patients^19,20^. Prostate cancer phenotype studies can be undertaken by observing expression profiles of various prostate cancer cell lines in prostate carcinomas. However, no systematic method is available for evaluating reference genes for either mRNA or miRNA expression in the two subtypes of prostate cancer cell lines (AR^+^, androgen-sensitive and AR^−^, androgen-insensitive).

Androgen is important in prostate cancer, with increasing evidences showing that mRNA and miRNA genes are disregulated when prostate cancer cells are treated with androgen^21–23^. For example, the percentage of transcriptomes of LNCaP cells directly or indirectly regulated by androgens is consistently between 1.5-4.3%^24^. Remarkably, these androgen-regulated genes contain reference genes^22^. Therefore, it is important to identify suitable reference genes in order to avoid erroneous normalisations in mRNA and miRNA expression studies for prostate cancer cell lines treated with androgens.

The overall aim of this study was to validate a set of suitable reference genes in prostate cell lines subjected to androgen treatment. To attain this aim, we obtained searched the literature to select 16 reference genes from published literature for mRNA or miRNA expression studies (see Supplementary Table S1). Sequentially, the expression stability of these reference genes was evaluated using three algorithms: geNorm, NormFinder, and Bestkeeper. The reference genes were sampled from a panel of five prostate cells including one human prostate epithelial cell and four prostate cancer cell lines treated with dihydrotestosterone (DHT), a testosterone metabolite. Finally, relative expression of target mRNA and miRNA, *PCA3* and *miR-141*, using the most/least stable reference genes, were used to validate the selection of candidate reference genes. The outcome of this study will be of benefit to qPCR based gene-expression studies in prostate cancer cells with/without DHT treatments.

## Results

### Validation of DHT influence on PSA expression

**Figure 1:**
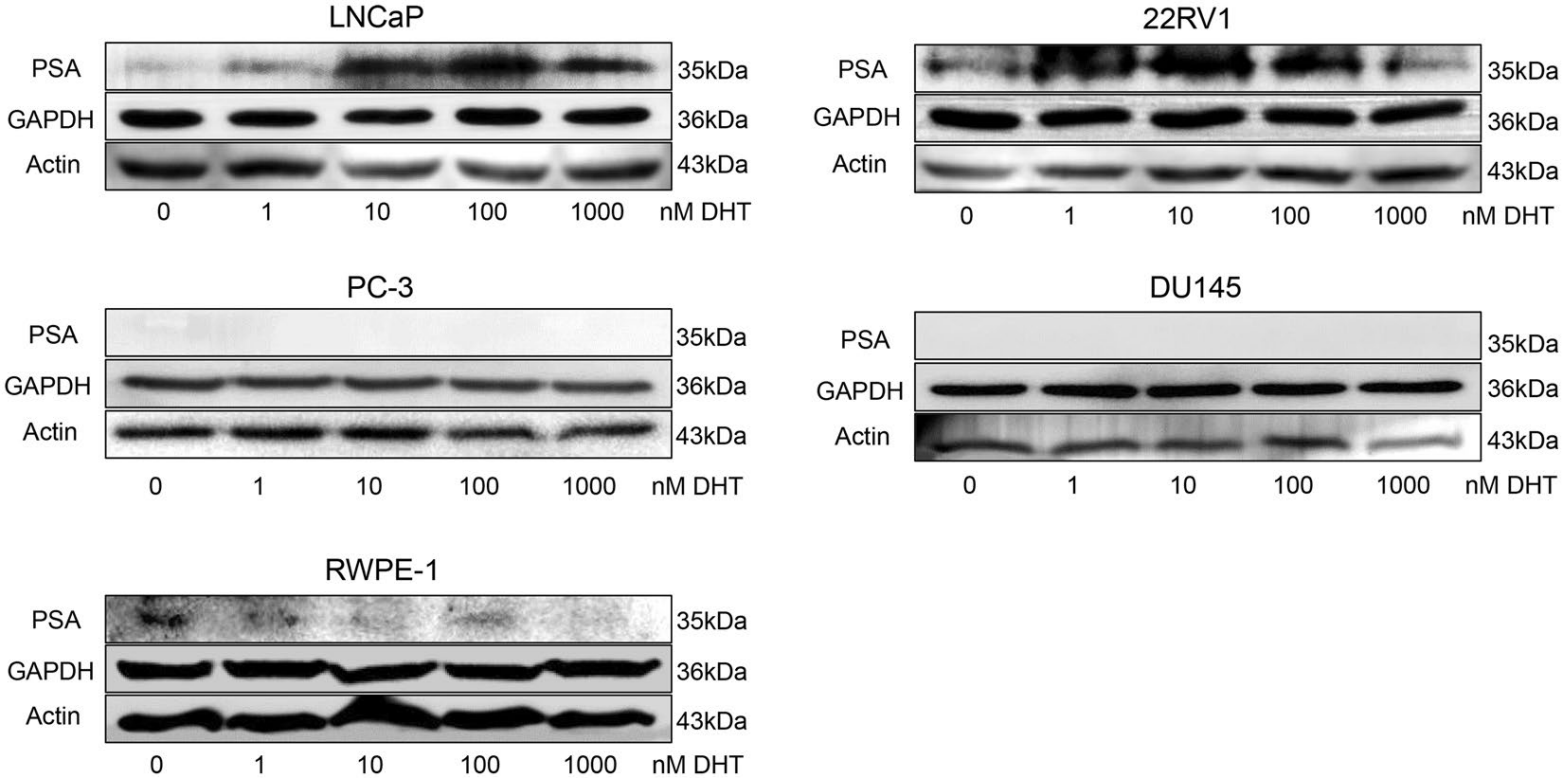
Validation of DHT treatments by Western blot. AR+ cells (LNCaP and 22RV1), AR − cells (DU145 and PC-3), and normal prostate epithelial cell (RWPE-1) were cultured using RPMI 1640 medium containing 10% charcoal-stripped FBS for 24 hours, and then treated with different DHT treatments. Validation of DHT treatments were performed through Western blot experiments using PSA as a marker, and GAPDH and Actin as control proteins. The grouping of blots were cropped from different parts of the same gel, or from different gels. Full-length unadjusted Western blot images for this figure as shown in Supplementary Fig. S3.

The AR is a ligand dependent transcription factor which regulates the expression of androgen-regulated genes. Prostate specific antigen (PSA, alias KLK3) is a well-known androgen-regulated gene^25^. Here, the effects of DHT treatments on prostate cell lines were validated by assessing the expression of PSA using Western blot experiments. Five cell lines were cultured using medium containing charcoal-stripped fetal bovine serum (FBS) for 24 hours, after which the cells were treated with different DHT concentrations. Clear PSA bands were detected in two AR+ cell lines (LNCaP and 22RV1), weak bands in normal prostate epithelial cell line (RWPE-1), but no band in either AR− cell lines (DU145 and PC-3 cells) (Fig. 1). As expected, the expression level of PSA was upregulated by DHT treatments in AR+ cell lines (Fig. 1). These outcomes validate the effects of DHT treatment on the AR+ cell lines in stripped serum experiments.

### RNA quality and qPCR assay validations

Stimulations experiments at different DHT concentrations were carried out on cells pre-cultured in medium with charcoal-stripped FBS. Total RNA was extracted from cell lines of 50 samples and evaluated for quality and integrity. Absorbance ratios of 260/280 nm and 260/230 nm, averaged (mean ± standard deviation) for all the five cell lines with different treatments, were 2.08 ± 0.02 and 1.74 ± 0.29, respectively (see Supplementary Table S2). RNA integrity numbers (RINs) ranged from 9.4 to 10 as detailed in Supplementary Table S2, indicating sufficient total RNA quality and integrity for the samples. All PCR assays produced single amplicons, indicated by the presence of single sharp amplification curves in qPCR reactions (see Supplementary Fig. S1). A confirmatory sequencing showed that each qPCR reaction was specific. For each candidate reference gene, correlation coefficients (R^2^) had a minimum value of 0.992, and efficiency values (E) were between 0.906 and 1.085 (inclusive) (Table 1).

**Table 1:** PCR efficiency for all primer pairs of reference genes and target genes. R^2^ indicates correlation coefficient.

Gene	R^2^	Efficiency	Slope
*18* *S rRNA*	0.998	0.906	−3.569
*ACTB*	0.997	1.065	−3.176
*ALAS1*	0.994	0.989	−3.346
*GAPDH*	0.997	0.924	−3.519
*HPRT1*	0.993	0.997	−3.330
*K-ALPHA-1*	0.998	0.987	−3.354
*RPL13A*	0.998	1.085	−3.134
*SDHA*	0.999	1.025	−3.263
*TBP*	0.995	1.025	−3.264
*YWHAZ*	0.997	1.009	−3.300
*PCA3*	0.993	0.979	−3.374
*miR-16*	0.999	0.917	−3.539
*miR-130b*	0.996	1.018	−3.280
*miR-1225-3p*	0.992	1.06	−3.186
*miR-1228-3p*	0.997	1.017	−3.283
*RNU-6-2*	0.998	0.982	−3.367
*RNU-43*	0.998	1.028	−3.257
*miR-141*	0.999	0.975	−3.383

### Expression profiles of candidate reference genes

**Figure 2:**
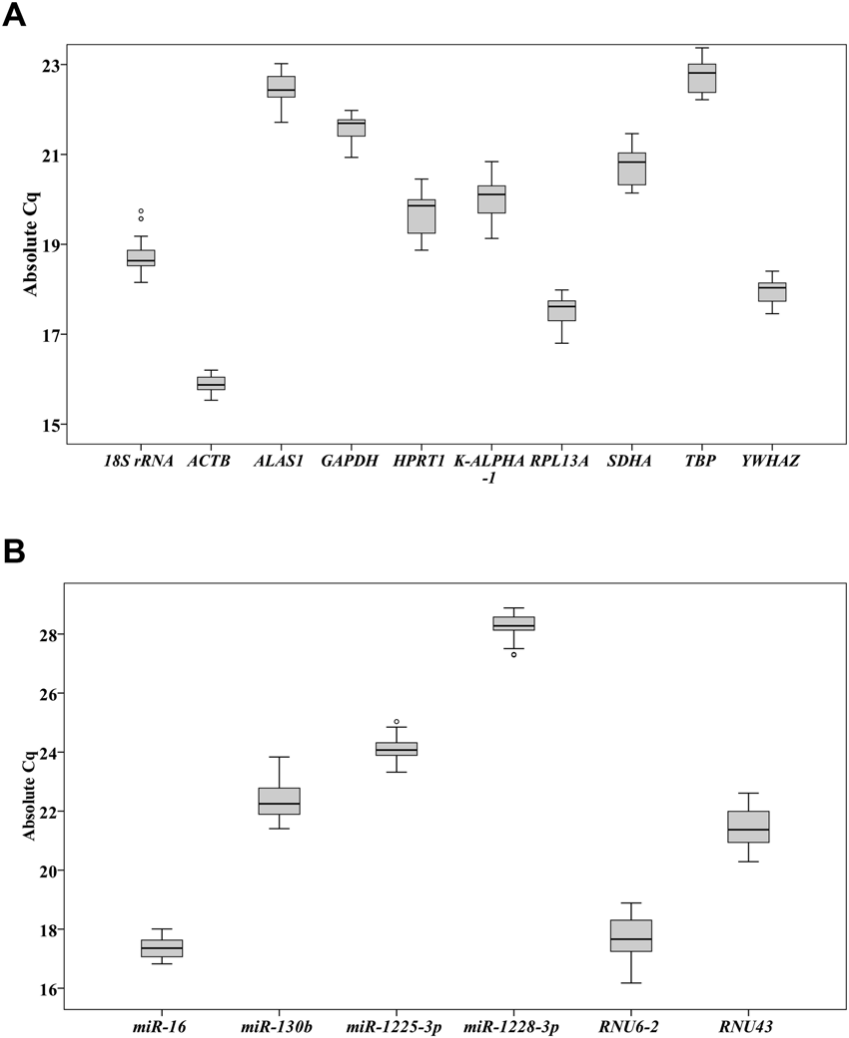
Box plots of absolute Cq values for each mRNA reference gene (**A**) and each npcRNA reference gene (**B**). Cells were cultured using RPMI 1640 medium containing 10% charcoal-stripped FBS for 24 hours, and then treated with different DHT concentrations. cDNA was prepared from these cells for the qPCR experiments. Expression of selected mRNA genes displayed as Cq across all prostate cell lines treated with various concentrations of DHT. The median is indicated by a line in each box, which in turn represents the 25^th^ and 75^th^ percentile. Whiskers indicate the 10/90 percentile ranges, and circles represent potential outliers.

We measured the expression of 10 mRNA reference genes and six non-protein coding RNA genes (npcRNA genes) (see Supplementary Table S1). Cq values of the 10 mRNA reference genes across all cell lines treated with different DHT concentrations ranged from 15 to 23 (Fig. 2A), indicating moderate abundance in the observed samples. Of all the 10 candidate genes, *ACTB* had the lowest mean Cq value (15.88 ± 0.21), indicating the highest expression, while *TBP* had the highest mean Cq (22.76 ± 0.39). In addition, within each cell line treated with different concentrations of DHT, Cq changes (ΔCq _DHT concentration_ = Cq _DHT concentration_ − Cq _DHT 0 nm_) for reference genes ranged from 0.00008 for *TBP* treated with 1 nM DHT in PC-3 cells to 0.651 for *K-ALPHA-1* treated with 1000 nM DHT in LNCaP (data not shown). The raw data suggest a considerably stable *ACTB* expression (Fig. 2A).

Expression of six npcRNAs reference genes for miRNA normalisation appears in Fig. 2B. The *miR-16* transcript was the most abundant with a mean Cq of 17.38 ± 0.35, while *miR-1228-3p* was the least abundant with a mean Cq of 28.25 ± 0.43. The raw data suggest that *miR-16* has stable expression, while the expression of *RNU6-2* and *RNU43* considerably vary across all cell lines under different DHT treatments.

### Identification of suitable reference genes

The most stable reference genes were identified with geNorm, NormFinder, and Bestkeeper algorithms. Each analysis is detailed in the following sections:

### geNorm analysis

**Figure 3:**
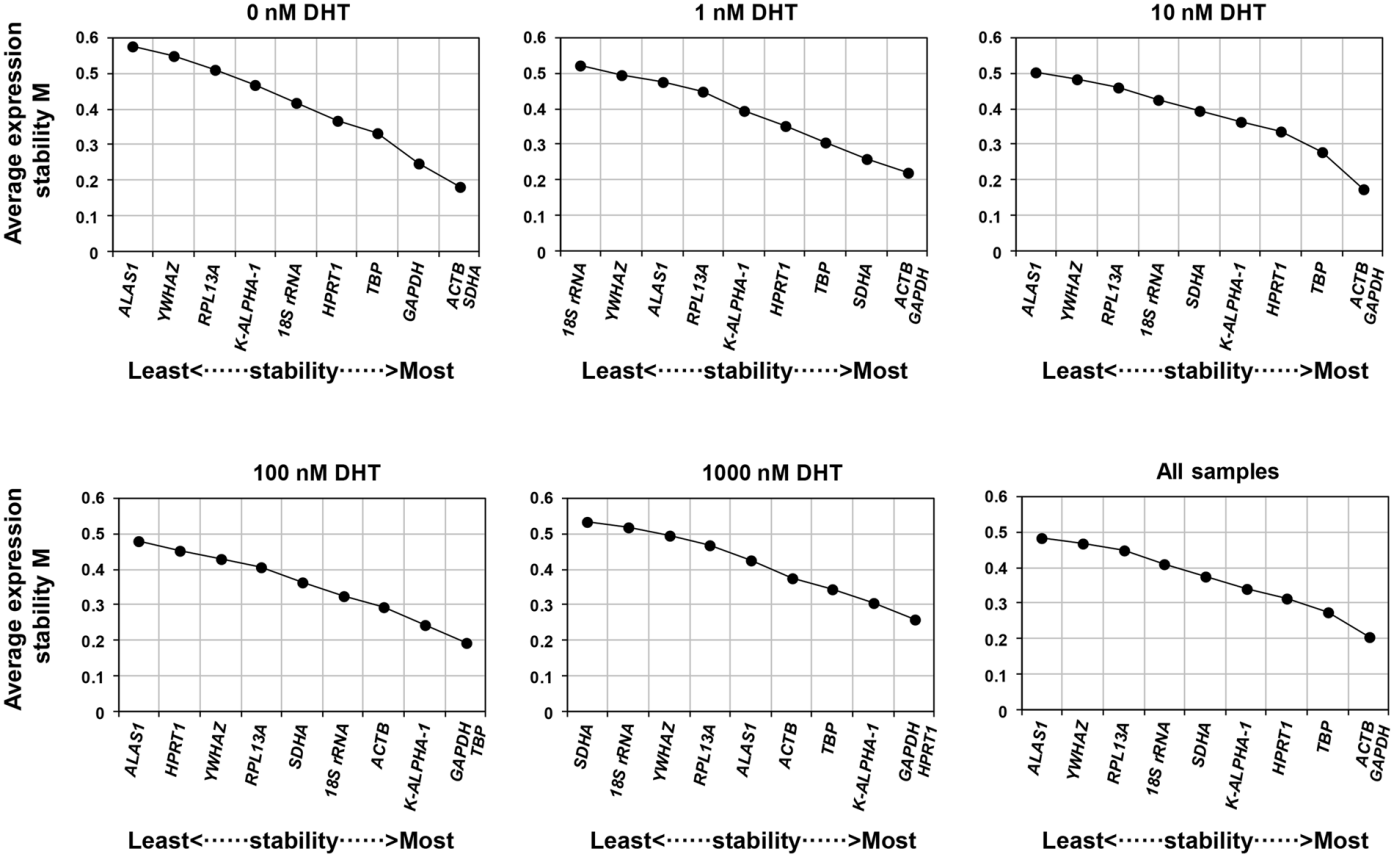
Expression stability and ranking of the ten reference genes as calculated by geNorm in various DHT treatments and all samples sets. Cells were cultured using RPMI 1640 medium containing 10% charcoal-stripped FBS for 24 hours prior to treatment with different DHT concentrations. cDNA was prepared from these cells and then used to perform qPCR experiments. The mean expression stability (M) was calculated by stepwise exclusion of the least stable gene by geNorm program. A lower M value indicates more stable expression. The least stable genes are presented on the left and the most stable on the right side of the plot.

**Figure 4:**
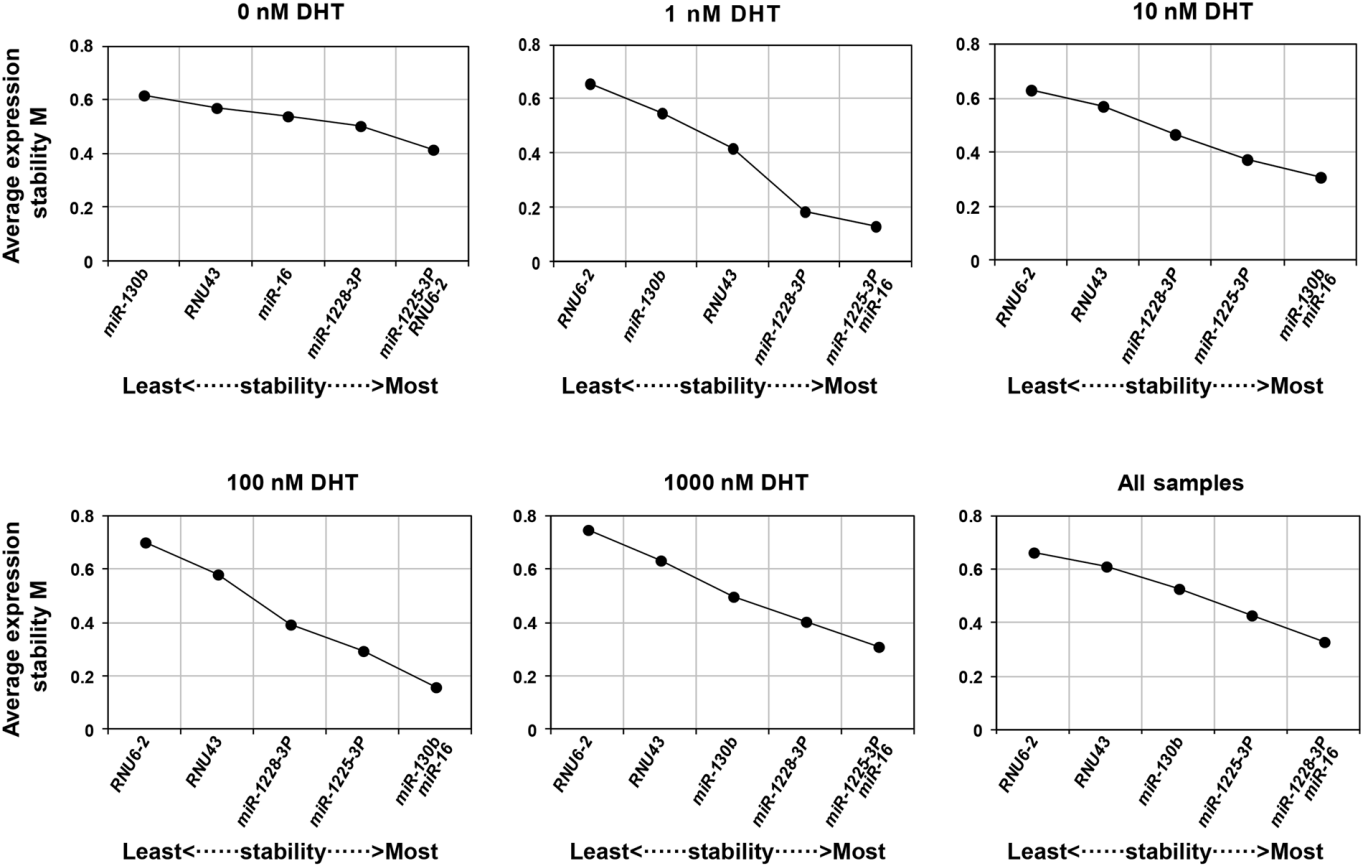
Expression stability and ranking of the six npcRNA reference genes as calculated by geNorm in various DHT treatments and all samples sets. Cells were cultured using RPMI 1640 medium containing 10% charcoal-stripped FBS for 24 hours prior to treatment with different DHT concentrations. cDNA for the qPCR experiments was prepared from these cells. The mean expression stability (M) was calculated by geNorm program. The least stable genes are presented on the left and the most stable on the right side of the plot.

Reference genes were ranked according to the expression stability value M^8^, with smaller M values signifying high gene expression stability. Our data showed that all the ten mRNA reference genes and six npcRNA genes exhibited high expression stability with low M values (<0.6) (Fig. 3) compared to the 1.5 stability threshold value in geNorm^8^. The rank order of the reference genes across the treatments indicated that *GAPDH* was the most stable reference gene except at 0 nM DHT treatment, while *ALAS1* was the most variable gene except at 1 nM DHT and 1000 nM DHT treatments. Among all the samples, *ACTB* and *GAPDH* had the lowest M value (0.207), followed by *TBP* and *HPRT1* with M values of 0.275 and 0.315, respectively (Fig. 3). In contrast, *ALAS1* exhibited the least stability (M value of 0.485) (Fig. 3). geNorm analysis outcomes for npcRNA genes stability are detailed in Fig. 4. The geNorm ranking showed that *miR-16* was the most stable gene, and *RNU6-2* the most variable gene except at 0 nM DHT treatment. Considering stability across all treatments, *miR-16* and *miR-1228-3p* were the most stable genes, while *RNU6-2* was the most variable gene.

**Figure 5:**
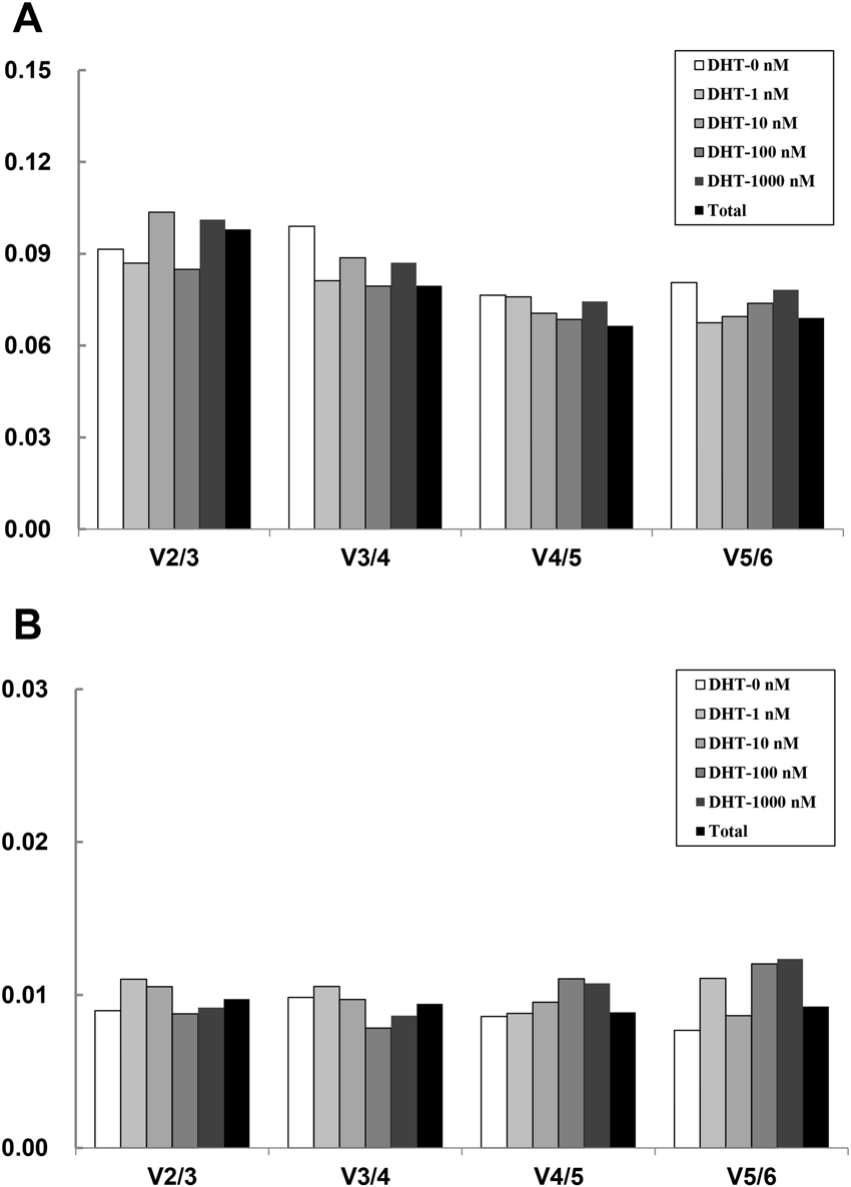
Determination of the optimal number of reference genes for normalisation. Cells were cultured using RPMI 1640 medium containing 10% charcoal-stripped FBS for 24 hours prior to treatment with different DHT concentrations. The obtained Cq values were analysed and a pair-wise variation value (Vn/n + 1) generated by geNorm analysis across all prostate cancer cell lines with different DHT concentrations for mRNA reference genes (**A**), and for miRNA reference genes (**B**).

geNorm analysis also calculates the optimal number of reference genes for gene expression analysis^8^. This occurs through stepwise calculation of the pairwise variation (V_n_/V_n+1_) between sequential normalisation factors (NF) (NF_n_/NF_n+1_) to determine the optimal number of genes required for geometric mean normalisation. A cut-off value of 0.15 for the pairwise variation was applied, below which there is no need to include additional reference genes^8^. As evident in Fig. 5, all pairwise variations V_2/3_ for mRNA and npcRNA reference genes were below 0.15, indicating that two reference genes combinations are sufficient for each treatment in this study.

### NormFinder analysis

In NormFinder analysis, reference genes with more stable expression are indicated by lower average expression stability values (SVs)^26^. For mRNA analysis, NormFinder ranking showed that *ACTB* and *GAPDH* had the most stable expression within all treatments across all cell lines (see Supplementary Table S3). For npcRNA analysis, the rank order of the reference genes across the treatments suggested that no single reference gene can be used ideally for all the treatments. For example, *miR-1228-3p* was identified as the most stable reference gene for 0 nM and 10 nM treatments, whereas *miR-1225-3p* was the best reference gene for 100 nM and 1000 nM treatments. With all the treatment conditions taken together, *ACTB* and *GAPDH*, and *miR-1225-3p* and *miR-16* were the most stable reference genes among the mRNA and npcRNA sets of reference genes, respectively.

### BestKeeper analysis

BestKeeper ranks the control reference genes on the basis of standard deviation (SD)^27^. The gene with lowest SD value is considered to be the most stable reference gene in terms of gene expression and vice versa. As shown in Supplementary Table S4, BestKeeper algorithm identified *ACTB* as the most suitable reference gene within all treatments. In contrast, either *HPRT1* or *K-ALPHA-1* had the least stability scores. In addition, BestKeeper ranking showed that *miR-16* was the most stable reference gene except at 10 nM and 100 nM DHT treatments.

Put together, these three programs showed some dissimilarity, potentially due to variation in their inherent strategies. Therefore, the geomean of ranking values was calculated to obtain a harmonised comprehensive ranking. As shown in Table 2, the comprehensive ranking indicates that *ACTB* and *GAPDH* were the most stable reference genes. The combination of *ACTB/GAPDH* formed the best candidate reference genes that can be used across all cell lines with different treatments for mRNA analysis (Table 2). In contrast, geNorm and NormFinder each identified *ALAS1* as the least suitable reference gene across most of treatments. On the other hand, geomean ranking revealed that *miR-16* and *miR-1228-3p* were the most suitable reference genes for npcRNA analysis. All the three strategies unanimously ranked *RNU6-2* as the least suitable reference gene for all cell lines.

**Table 2:** Stability ranking of 10 mRNA reference genes and 6 npcRNA reference genes analysed by three algorithms across all treatments.

	geNorm		NormFinder		BestKeeper		Recommended comprehensive ranking	
	Gene name	Ranking	Gene name	Ranking	Gene name	Ranking	Gene name	Geomean of ranking values
mRNA gene	*ACTB*	1	*ACTB*	1	*ACTB*	1	*ACTB*	1.00
	*GAPDH*	1	*GAPDH*	2	*YWHAZ*	2	*GAPDH*	2.00
	*TBP*	3	*TBP*	3	*RPL13A*	3	*TBP*	3.98
	*HPRT1*	4	*HPRT1*	4	*GAPDH*	4	*RPL13A*	5.24
	*K-ALPHA-1*	5	*SDHA*	5	*18 S rRNA*	5	*HPRT1*	5.43
	*SDHA*	6	*RPL13A*	6	*ALAS1*	6	*YWHAZ*	5.45
	*18 S rRNA*	7	*K-ALPHA-1*	7	*TBP*	7	*SDHA*	6.21
	*RPL13A*	8	*18 S rRNA*	8	*SDHA*	8	*18 S rRNA*	6.54
	*YWHAZ*	9	*YWHAZ*	9	*K-ALPHA-1*	9	*K-ALPHA-1*	6.80
	*ALAS1*	10	*ALAS1*	10	*HPRT1*	10	*ALAS1*	8.43
npcRNA gene	*miR-16*	1	*miR-1225-3p*	1	*miR-16*	1	*miR-16*	1.26
	*miR-1228-3p*	1	*miR-16*	2	*miR-1228-3p*	2	*miR-1228-3p*	1.82
	*miR-1225-3p*	3	*miR-1228-3p*	3	*miR-1225-3p*	3	*miR-1225-3p*	2.08
	*miR-130b*	4	*miR-130b*	4	*RNU43*	4	*miR-130b*	4.31
	*RNU43*	5	*RNU43*	5	*miR-130b*	5	*RNU43*	4.64
	*RNU6-2*	6	*RNU6-2*	6	*RNU6-2*	6	*RNU6-2*	6.00

### Reference gene stability in AR+/AR− cell lines

To investigate DHT effects on different cell types, cell lines were divided into AR+ cells (LNCaP and 22RV1) on one hand, and AR− cells and normal cell lines (DU145, PC-3 and RWPE-1) on the other hand. Data from these studies appear in Table 3, which describes the recommended comprehensive ranking based on geNorm, NormFinder, and BestKeeper analyses, and indicates stability ranking of candidate genes having different patterns between the two groups. In addition to treatment with 10 nM DHT, there were different combinations of the two stable reference genes between the two groups under similar DHT treatments. We observed that after DHT treatments, stability rankings dramatically fluctuated in the AR+ group more than in the AR− and normal cell group. For example, in the AR+ cell lines, stability rankings of *K-ALPHA-1* changed from the seventh position without DHT treatment to the first position after 100 nM DHT treatment. Finally, *ACTB/GAPDH* and *ACTB/HPRT1* were identified as the most stable reference gene combinations in AR+ group, and AR− and normal cell lines group, respectively. Assessing the stability of npcRNA genes, we also noted a cell group dependent variation of stability scores. *RNU6-2* was among the most stable reference gene in AR− and normal cell lines, but dropped to the bottom rank in the AR+ cell lines irrespective of DHT concentrations (Table 3). Put together, our results suggest that *miR-16* and *miR-1228-3p* is the most suitable reference gene combination in AR+ cell line, while *RNU6-2* and *RNU43* combination stands out for AR− and normal cell lines (Table 3).

**Table 3:** Stability ranking of reference genes using geomean of ranking values analysed by three algorithms when prostate cells are treated with different DHT concentrations.

	Group	Gene	All treatments	0 nM	1 nM	10 nM	100 nM	1000 nM
mRNAgene	AR+celllines	*ACTB*	1	1	3	2	5	1
		*GAPDH*	2	4	6	3	4	5
		*YWHAZ*	3	2	1	4	3	3
		*SDHA*	4	5	2	1	2	4
		*TBP*	5	3	4	6	6	6
		*K-ALPHA-1*	6	7	8	5	1	8
		*18 S rRNA*	7	6	5	9	9	9
		*ALAS1*	8	8	7	7	7	2
		*RPL13A*	9	9	9	8	8	7
		*HPRT1*	10	10	10	10	10	10
	The other cell lines	*ACTB*	1	1	5	1	2	1
		*HPRT1*	2	4	1	4	3	2
		*SDHA*	3	5	3	2	1	4
		*RPL13A*	4	6	6	3	5	5
		*TBP*	5	2	2	6	6	3
		*ALAS1*	6	3	4	7	8	7
		*K-ALPHA-1*	7	8	7	8	4	8
		*YWHAZ*	8	7	8	9	9	6
		*GAPDH*	9	9	9	5	10	10
		*18 S rRNA*	10	10	10	10	7	9
npcRNAgene	AR+celllines	*miR-16*	1	2	1	2	3	1
		*miR-1228-3p*	2	3	2	1	1	3
		*miR-1225-3p*	3	1	4	3	2	2
		*RNU43*	4	5	5	5	4	6
		*miR-130b*	5	6	3	4	5	5
		*RNU6-2*	6	4	6	6	6	4
	The other cell lines	*RNU6-2*	1	1	2	2	1	2
		*RNU43*	2	2	1	1	3	1
		*miR-1228-3p*	3	5	4	3	4	6
		*miR-16*	4	3	3	4	2	4
		*miR-1225-3p*	5	4	6	6	5	3
		*miR-130b*	6	6	5	5	6	5

Cell lines were divided into two groups: AR+ cell lines (LNCaP and 22RV1), and AR− and normal cell lines (DU145, PC-3 and RWPE-1).

### Validation of the reference genes

**Figure 6:**
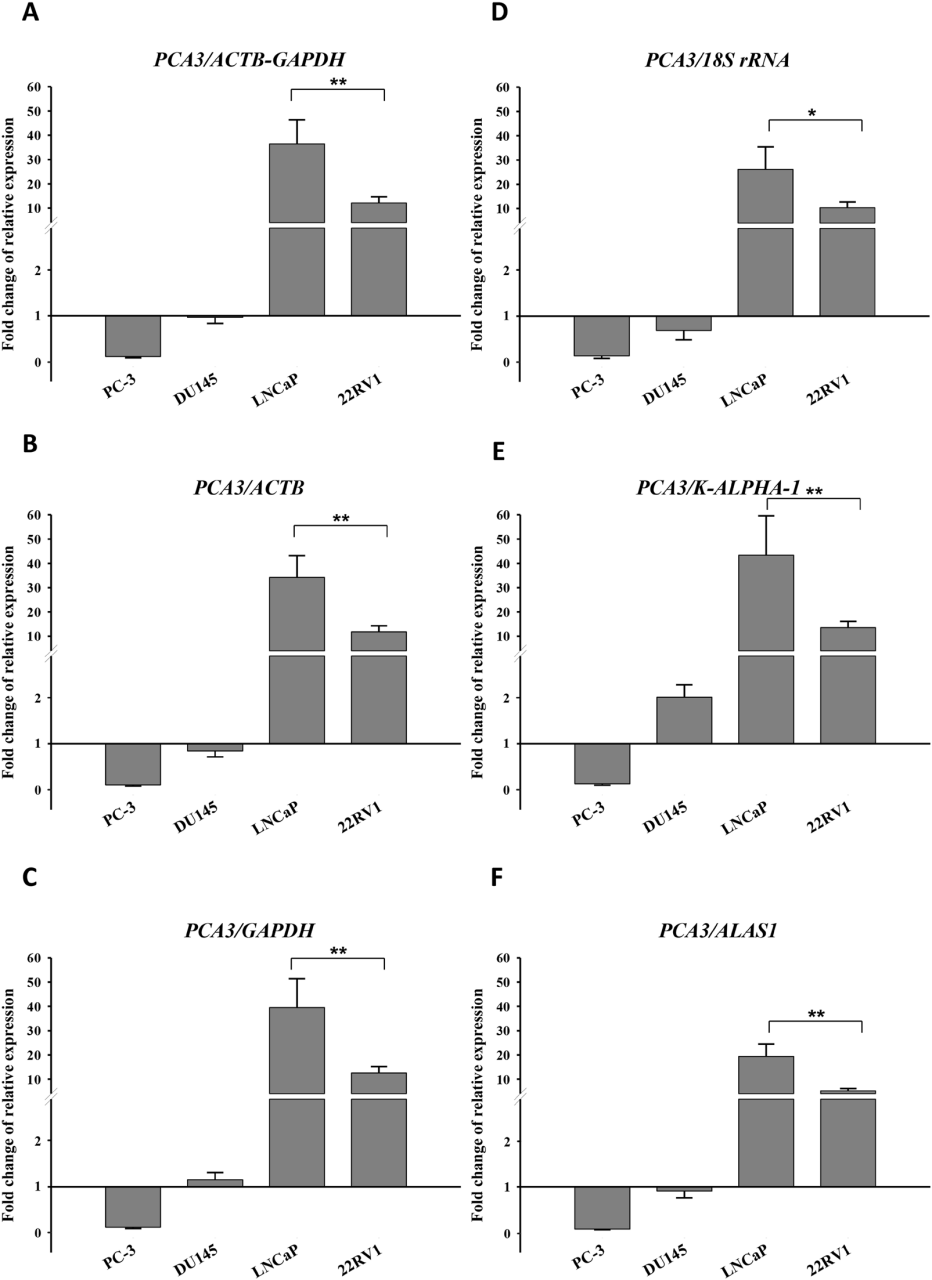
Relative quantification of *PCA3* expression depends on mRNA reference gene. Cells were cultured using RPMI 1640 medium containing 10% charcoal-stripped FBS for 24 hours prior to treatment with different DHT concentrations. The cDNA was prepared from these cells for the amplification of *PCA3* and mRNA reference genes by qPCR. Relative expression of *PCA3* across all cell lines was normalised by the best reference genes combination (*ACTB-GAPDH*) (**A**), by the most stable single gene *ACTB* (**B**) or *GAPDH* (**C**), by the least stable single gene *18 S rRNA* (**D**), or *K-ALPHA-1* (**E**), or *ALAS1* (**F**). Y-axis indicates the fold change of relative expression of *PCA3* in RWPE-1 cells set to 1. Error bars indicate the standard error (±SE) evaluated from three biological replicates. * and **Indicate P < 0.05 and P < 0.01, respectively.

In order to validate the selection of reference genes, we tested reference gene efficiency on target gene quantification using *PCA3* as a target gene. *PCA3*, also called *DD3*, is a prostate-specific gene that is highly overexpressed in prostate cancer. It is popularly used as a sensitive and specific marker for prostate tumour detection^14,28,29^. Similar to a previous report^14^, we observed a substantial overexpression of *PCA3* in AR+ cell lines compared to AR− cell lines (Fig. 6). *PCA3* expression normalised by the most stable reference genes, *ACTB* and *GAPDH*, singly and in combination, exhibited similar high-low patterns (Fig. 6A–C). In contrast, *PCA3* expression exhibited uneven patterns when normalised by the least stable reference genes (Fig. 6D–F). For example, the fold-change of *PCA3* expression in LNCaP cells was approximately 38 when normalised by the suitable reference genes (Fig. 6A–C) but remained less than 20 when normalised using *ALAS1* (Fig. 6F) or approximately 25 using 18* S rRNA* (Fig. 6D). Moreover, the *PCA3* expression in DU145 cells was significantly higher (P < 0.001) than in RWPE-1 cells when normalised by *K-ALPHA-1* (Fig. 6E), but no difference in expression was found between the two cell lines when normalised by the most stable reference genes (Fig. 6A–C). Additional *PCA3* expression profiles in LNCaP cells treated with various DHT concentrations are shown in Figure S2. Although the effect of DHT treatment on *PCA3* expression was validated, higher fold changes of *PCA3* expression were observed in normalisations using the least stable reference gene (*K-ALPHA-1*) compared to the most stable reference genes (*ACTB* and *GAPDH*) or their combination. Additionally, significant differences in *PCA3* expression were found between 1 nM DHT and 100 nM/1000 nM DHT treatments normalised by the suitable references, while no statistical difference in expressional levels was found among different DHT treatments normalised by *ALAS1*.

**Figure 7:**
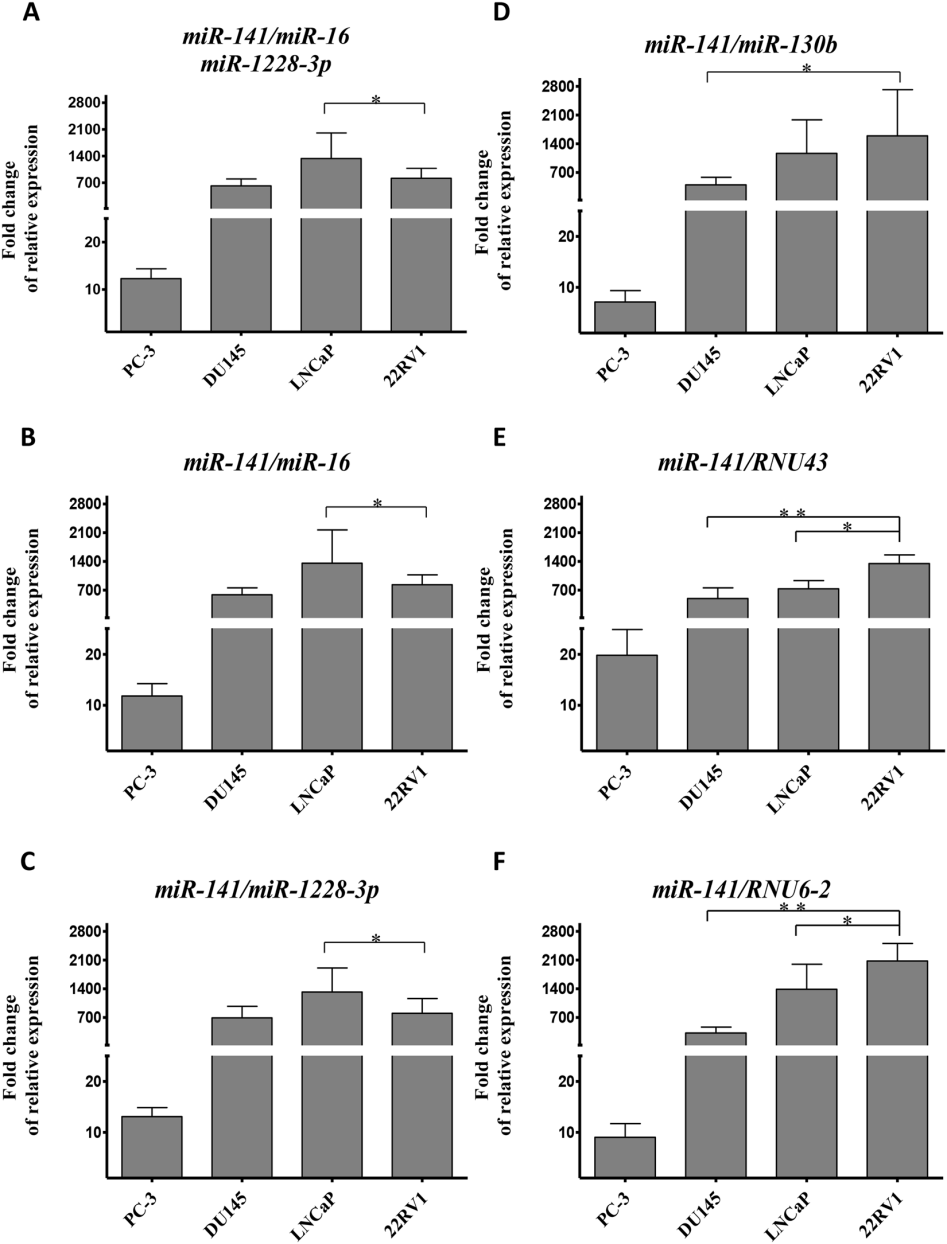
Relative quantification of *miR-141* expression depends on different npcRNA reference genes. Cells were cultured using RPMI 1640 medium containing 10% charcoal-stripping FBS for 24 hours prior to treatment with different DHT concentrations. The cDNA was prepared from these cells for the amplification of *miR-141* and npcRNA reference genes by qPCR. Relative expression of *miR-141* across all cell lines was normalised with the best reference genes combination (*miR-16-miR-1228-3p*) (**A**), by the most stable single gene *miR-16* (**B**) or *miR-1228-3p* (**C**), by the least stable single gene *miR-130b* (**D**), or *RNU43* (**E**), or *RNU6-2* (**F**). Y-axis indicates the fold change of relative expression of *miR-141* in RWPE-1 cells set to 1. Error bars indicate the standard error (± SE) evaluated from three biological replicates. * and **Indicate P < 0.05 and P < 0.01, respectively.

To validate reference genes for measuring miRNA expression, we choose *miR-141* as a target gene. Previous work indicates that *miR-141* is overexpressed in prostate cancer and CRPC compared to benign prostate hyperplasia^30^. Relative expression of *miR-141* across all cell lines under various treatments is presented in Fig. 7. *miR-141* expression was similar across normalisations carried out using the most stable npcRNA genes (*miR-16* and *miR-1228-3p*), both singly and in combination. However, using the least stable reference genes (*miR-130b*, *RNU43*, or *RNU6-2*) to normalise the quantification of *miR-141*, relative expression of *miR-141* varied across all cell lines. There was a stark contrast in the expression patterns of *miR-141* in LNCaP cells under different normalisation treatments. For instance, *miR-141* expression was highest in LNCaP cells under normalisation with the most stable reference genes (Fig. 7A–C), but lower under the least stable reference genes (Fig. 7D–F). *miR-141* expression was significantly lower in LNCaP than in 22RV1 cell lines when normalised by *RNU43* (p = 0.013) or *RNU6-2* (p = 0.045) (Fig. 7E and F).

## Discussion

Androgens play an important role in the growth of both androgen dependent prostate cancer and CRPC. After the binding of androgen to AR, AR complex changes in conformation and composition in the cytoplasm. This change leads to the translocation of AR into the nucleus and its subsequent binding to androgen response elements (AREs) in the promoter and enhancer regions of various target genes^31^. As a result, many genes are involved in dysregulation by androgen^24^. Therefore, the identification of androgen-regulated genes is of great importance for discovering the mechanisms behind development and progression of prostate cancer. Importantly, to obtain accurate expression results of target genes, it is necessary to validate a set of suitable reference genes under varied experimental conditions.

To our knowledge, this is the first identification of reference genes for mRNA and miRNA normalisation using qPCR technology in normal prostate and cancer cell lines following various DHT treatments. Koramutla *et al*. have emphasised the importance of validating treatment-effects in test samples before RT-qPCR experiments^32^. Thus, in this study, experiments for validating the effect of DHT treatment on prostate cells were first performed by evaluating marker protein expression (Fig. 1). Then, ten mRNA and six npcRNA candidate genes were assessed for their stability as reference genes. Our results have shown that stability rankings of these genes dramatically fluctuated after DHT treatments, especially in the AR+ cell lines (Table 3). Considering all treatments with various concentrations of DHT, the recommended comprehensive ranking computed by three methods (geNorm, NormFinder, and Bestkeeper) identified *ACTB* and *GAPDH* as the most stable reference gene candidates for mRNA analysis in all the investigated cell lines. This concurs with previous reports in primary culture of prostate cancer cells and human formalin fixed paraffin embedded tissue samples of prostate cancer^12,15^. Another previous gene expression study in prostate cancer tissue indicated that *HPRT1*, *ALAS1*, and *K-ALPHA-1* were the most stable reference genes^13^. However, our analysis showed that *ALAS1* and *K-ALPHA-1* had the least stability as reference genes (Table 2), and *HPRT1* was the least suitable reference gene in AR+ cell lines (Table 3). The conflicting observations could be explained by differences in sample sources, as the previous study used tissue samples^13^ in contrast to our study which used cell lines treated with androgens. We further found that stability rankings of *HPRT1* depended on cell type. *HPRT1* was ranked in the first four positions in AR− and normal cells group, but was at the bottom position in the AR^+^ group (Table 3). For npcRNA reference genes, *miR-16*, *miR-1228-3p*, and *miR-1225-3p* were identified by the three algorithms (geNorm, NormFinder, and Bestkeeper) to be the most stable reference genes.

Normalisation factors generated by two genes (*miR-16* and *miR-1228-3p*) could be suitable for improving the reliability of expression quantification. Notably, *miR-16* has been highly regarded as the most suitable reference gene in several cancer studies including colorectal cancer^33^, breast cancer^34,35^, gastric cancer^36^, and bladder cancer^37^. Our data showed that *RNU6-2*, which is the most frequently used reference gene in studies, was the least stable reference gene for all prostate cell lines (Table 2), especially in AR+ cell lines (Table 3). Similar finding has been reported for miRNA expression in prostate cancer tissues^17^, urothelial carcinomas^38^, cervical carcinogenesis^39^, and serum^40^. *RNU6-2* (*RNU6B*) is a small nucleolar RNA (snoRNA) that forms part of the *RNU6* small nuclear ribonucleoprotein. Because of its large size, it is likely to be degraded. Hence *RNU6-2* expression is reportedly less stable than miRNA^41^.

To validate the selected reference genes, we measured relative expression of *PCA3* and *miR-141* and noted that inappropriate use of reference genes could considerably impact the results, not only of mRNA expression (Figs 6 and S2), but also of miRNA expression (Fig. 7). Thus selecting suitable reference genes is important when quantifying both mRNA and miRNA with qPCR.

In conclusion, our work provides a foundation for translational mRNA and miRNA research for prostate cancer cell lines with DHT treatments. Analysis of expression stability using geNorm, NormFinder, and BestKeeper revealed that *ACTB* and *GAPDH*, and *miR-16* and *miR-1228-3p* were appropriate reference genes for mRNA and miRNA normalisation, respectively. Considering prostate cancer cell types, *ACTB /GAPDH* and *ACTB/HPRT1* are suggested to be the most suitable reference gene combinations for mRNA analysis. On the other hand, *miR-16/miR-1228-3p* showed the best performance for miRNA analysis in AR+ cell lines, while *RNU6-2/RNU43* was the best in AR− and normal cell lines. These findings have important implications for translational research of prostate cancer cells with different DHT treatments using RT-qPCR technology.

## Materials and Methods

### Cell culture and androgen treatment

One normal prostate epithelial cell line (RWPE-1) and four prostate cancer cell lines (AR+, LNCaP and 22RV1; AR−, DU145 and PC-3) were used. All cell lines were from American Type Culture Collection (ATCC, www.atcc.org, Manassas, VA, USA). RWPE-1 cells were cultured in DMEM high glucose (HyClone, Utah, USA), with 4.5 g/L glucose, 4 mM L-glutamine containing 10% FBS (Gibco, Carlsbad, CA, USA), 100 units/mL penicillin, and 100 µg/mL streptomycin. Four prostate cancer cell lines were cultured in RPMI 1640 Medium (HyClone) with 10% FBS (Gibco), 100 units/mL penicillin and 100 µg/mL streptomycin. Cells were maintained in a humidified atmosphere with 5% CO_2_ at 37 °C.

Considering that 1 nM, 10 nM, 100 nM, and 1000 nM DHT concentrations are usually used in DHT stimulation studies, we selected the four concentrations to treat prostate cell lines plus 0 nM DHT as control. In detail, cells were seeded at a density of 1 × 10^5^ cells in 6-well plates and cultured for 24 hours in RPMI 1640 medium containing 10% charcoal-stripped FBS (Biological Industries, Kibbutz Beit-Haemek, Israel). The cells were subsequently treated with 1, 10, 100, and 1000 nM DHT (Sigma, Buchs, Switzerland) dissolved in ethanol, or ethanol carrier alone, and incubated for 48 h. The final ethanol concentration in media was 0.01%. Cells were then lysed with qiazol reagent (Qiagen, Hilden, Germany). These experiments were performed in duplicate.

### Selection of candidate reference genes

A total of 10 protein coding genes (mRNA genes) and six non-protein coding genes (npcRNA genes) were selected for expression analysis based on a search of relevant literature, particularly those relating to reference genes previously identified in prostate cancer. Gene characteristics are summarised in Table 1. The candidate reference genes are distributed among different chromosomes and provide variant molecular functions. Among these candidate mRNA genes, *ACTB*, *ALAS1*, *GAPDH*, *HPRT1*, *K-ALPHA-1*, *RPL13A*, *SDHA*, and *TBP* have been reported to be optimal reference genes for normalisation in prostate cancer tumor and normal tissues^12–14^, or in primary culture of prostate cancer cells^15^. In addition, the *18 S rRNA* gene was identified as a valid reference gene for expression studies in human breast cancer cell lines^42^. The *YWHAZ* gene is a commonly used reference gene. For miRNA expression studies, four miRNAs (*miR-16*, *miR-130b*, *miR-1225-3p*, and *miR-1228-3p*) and two small nucleolar RNA genes (*RNU6-2* and *RNU43*) were selected as candidate npcRNA reference genes (Table S1). Two of the npcRNA genes (*miR-130b* and *RNU6-2*) have been identified as stable reference genes for miRNA normalisation in prostate cancer tissue^18^, three (*miR-1225-3p, miR-1228-3p*, and *RNU43*) as optimal reference genes for several cancer patients with prostate cancer^19,20^, while *miR-16* is reported to be an optimal reference gene for several cancers^33–37^.

### Western blot for validating treatment effect

To validate the effect of DHT-treatments on prostate cell lines, cells were first cultured in RPMI 1640 medium containing 10% charcoal-stripped FBS for 24 hours, and then treated with different DHT concentrations. The cells were then dissolved by radioimmunoprecipitation assay (RIPA) buffer (Pierce, IL, USA), with the addition of EDTA-free protease inhibitor Cocktail Tablets (Roche, Mannheim, Germany). Protein concentration in the supernatant was determined by the bicinchoninic acid (BCA) protein assay kit (Thermo, IL, USA). The cell lysate was subjected to SDS-PAGE and transferred onto polyvinylidene fluoride (PVDF) membranes (Millipore, MA, USA). The membranes were incubated with anti-PSA monoclonal antibody (1:1000, Cell Signaling Technology, MA, USA) or anti-Actin monoclonal antibody (1:1000, Cell Signaling Technology, MA, USA), or anti-GAPDH monoclonal antibody (1:2000, Beyotime Biotechnology, Shanghai, China) and then incubated with horseradish peroxidase-conjugated secondary antibody (1:5000, Santa Cruz Biotechnology Inc. California, USA). Immunoreactive bands were visualised by SuperSignal West Pico Chemiluminescent Substrate (Thermo, IL, USA) using Amersham Imager 600 (GE, Tokyo, Japan).

### RNA extraction and cDNA synthesis

Total RNA extraction, including DNase treatment with RNase-free DNase I set (TianGen, Beijing, China), was carried out using an miRNeasy Mini Kit (Qiagen, Hilden, Germany) according to the manufacturer’s instructions. Extracted RNA was quantified by NanoDrop 2000 Spectrophotometer (Thermo Fisher Scientific, Roskilde, Denmark), and the absorbance ratios at 260/280 nm and 260/230 nm were measured to assure RNA purity. RNA samples were then assessed with an RNA 6000 Nano kit (Agilent Technologies, Waldbronn, Germany) using the Agilent 2100 electrophoresis Bioanalyzer (Agilent Technologies, CA, USA) to obtain an RIN.

Total RNA (1 or 2 μg) was reverse-transcribed using the miScript II RT Kit (Qiagen) with miScript HiFlex buffer or miScript HiSpec buffer, and the cDNA generated was used as a template for quantification of mRNA and miRNA respectively.

### Quantitative PCR (qPCR)

qPCR was performed on 16 putative reference genes and two target genes (*PCA3* and *miR-141*). Primer details for the mRNA study are summarised in Supplementary Table S5. Primer specificity was confirmed for each primer set using UCSC’s human genome browser (http://genome.ucsc.edu) and the Primer-BLAST at National Centre for Biotechnology (NCBI) browser (http://www.ncbi.nlm.nih.gov). Primers for miRNA study were purchased from miScript primer assay (Qiagen). qPCR reactions were conducted in a 96-well plate using ABI PRISM 7500 Real-Time system (Applied Biosystems, Foster, CA, USA). Each reaction was performed in triplicate. For mRNA quantification, each reaction had a 20-µL volume, containing 1 × SYBR® Premix Ex Taq™ II (Takara Biotechnology, Dalian, China) and 1 µL cDNA. Cycling conditions were as follows: 95 °C for 10 s, followed by 40 cycles of 95 °C for 5 s and 60 °C for 31 s. For miRNA quantification, each reaction was performed in a 20-µL volume containing 1 × QuantiTect SYBR Green PCR Master Mix (Qiagen), 2 µL of each PCR primer and 1 µL cDNA. Reaction conditions were as follows: 95 °C for 15 min, followed by 40 cycles of 94 °C for 15 s, 55 °C for 30 s, and 70 °C for 30 s.

PCR reaction specificity was confirmed with a DNA melting curve analysis and gel electrophoresis of products. PCR products were further cloned into PMD18-T vector (Takara Biotechnology) and sequenced. Each experiment included a no-template control and a cDNA standard curve for each gene. The amplification efficiency for each primer pair was determined using the formula E = [10^(−1/slope)^ − 1], using the slope of the semi-log regression plot of Cq versus log input of cDNA (10-fold dilution series of five points from the LNCaP cells).

### Data analysis

Candidate reference gene stability was evaluated with geNorm^8^, NormFinder^26^, and BestKeeper^27^. geNorm and NormFinder calculations are based on converted quantities according to the formula: 2^−ΔCt^ (ΔCt = the corresponding Cq value-minimum Cq). BestKeeper calculates the standard deviation (SD) and intra-run coefficient of variation (CV) based on raw Cq values. From the ranks observed from each program, we calculated the geometric mean of their weights for the final ranking. The values of relative normalised expression were calculated for each target gene using the 2^ΔΔCt^ method^43^. Comparison of means was done by Student’s t test using SPSS v.22.0 software (IBM).

## Electronic supplementary material

Supplementary information
