# Supplementary material for: Identification of valid reference genes for mRNA and microRNA normalisation in prostate cancer cell lines
Source: Sci Rep. 2018 Jan 31;8:1949. doi: 10.1038/s41598-018-19458-z (PMC5792445; doi:10.1038/s41598-018-19458-z)
Supplement: Supplementary file 1 — Supplementary information [file 41598_2018_19458_MOESM1_ESM.pdf]

Identification of valid reference genes for mRNA and microRNA normalisation in prostate cancer cell lines

Hui Zhao[1, 2#], Teng-Fei Ma[1,3 #], Jie Lin[3 #],Lin-Lin Liu[1,3], Wei-Jie Sun[1,3], Li-Xia Guo[1,3], Si-Qi Wang[1,3], Newton O. Otecko[4,5], Ya-Ping Zhang[1, 4]*

Supporting Information

Figure S1. Melting peaks of all primer pairs. The specificity of all the primer pairs was confirmed by a single peak in the melting curve.

Figure S2. Relative expression of *PCA3* normalised by different reference genes and their combinations. Fold change in *PCA3* expression of LNCaP cells subjected to different DHT treatments. Stable reference genes *YWHAZ*, *ACTB*, their combination, and least stable reference genes *SDHA* and *ALAS1* were independently used for normalisation. Bars indicate +SD evaluated from three biological replicates. * and ** indicate $P<0.05$ and $P<0.01$, respectively.

Figure S3. Full-length unadjusted images of Western blots shown in Figure 1.

Table S1. Information on mRNA and npcRNA reference genes used in this study. * indicates *Homo sapiens* unplaced genomic contig, GRCh37.p5.

**Gene symbol**	**Gene name/RNA Species**	**Chromosomal Localisation**	**Molecular function**
18S rRNA	18S ribosomal RNA	ChrUn*	Ribosome subunit, translation
ACTB	β-Actin	7p22-p12	Cytoskeleton
ALAS1	aminolevulinate, delta-, synthase 1	3p21.1	Heme synthesis
GAPDH	Glyceraldehyde-3-phosphate dehydrogenase	12p13.31	Glycolysis
HPRT1	Hypoxanthine phosphoribosyl-transferase 1	Xq26.1	Generation of purine nucleotides
K-ALPHA-1	K-alpha-1 tubulin, alpha, ubiquitous	12q13.12	Structural constituent of cytoskeleton
RPL13A	Ribosomal protein L13a	19q13.3	Catalyze protein synthesis
SDHA	Succinate dehydrogenase complex, subunit A	5p15	Glycolysis
TBP	TATA box binding protein	6q27	Transcription initiation
YWHAZ	Tyrosine 3-monooxygenase	8q23.1	Signal transduction
miR-16	miRNA	13q14.2	Negative regulation in chronic lymphocytic leukemia
miR-130b	miRNA	22q11.21	Gene expression regulation in cell growth and cell invasion
miR-1225-3p	miRNA	16p13.3	Gene expression regulation in stem cell self-renewal and differentiation
miR-1228-3p	miRNA	12q13.3	Cellular development and lipid Metabolism
RNU6-2	snoRNA*	19p13.3	Cellular RNAs modification
RNU43	snoRNA	22q13.1	Ribosomal RNA modification

Table S2. Quantity and integrity of total RNA in this study.

		Treatment I				Treatment II			
	DHT (nM)	RNA conc. (ng/ul)	260/280	260/230	RIN	RNA conc. (ng/ul)	260/280	260/230	RIN
RWPE-1	0	760	2.08	1.46	10	712	2.06	2.11	10
	1	692	2.09	1.66	9.9	628	2.09	1.47	10
	10	716	2.06	2.12	10	733	2.10	1.59	10
	100	653	2.08	1.42	10	688	2.10	1.52	9.8
	1000	630	2.08	1.85	10	627	2.09	1.48	10
LNCaP	0	506	2.05	2.09	9.9	519	2.02	1.56	9.8
	1	536	2.07	1.41	9.8	454	2.04	1.89	9.9
	10	559	2.06	2.00	9.7	488	2.02	1.93	10
	100	576	2.08	1.66	9.8	455	2.05	1.53	9.6
	1000	582	2.08	2.04	9.9	410	2.05	1.90	9.7
DU145	0	432	2.06	1.91	10	390	2.10	0.92	9.6
	1	487	2.05	1.58	9.7	356	2.09	1.42	9.8
	10	420	2.05	2.05	9.6	375	2.08	1.90	9.9
	100	402	2.07	2.04	9.7	364	2.08	2.05	9.6
	1000	516	2.02	1.90	9.8	359	2.08	1.82	9.5
22RV1	0	503	2.08	1.98	10	423	2.10	2.03	10
	1	447	2.11	1.80	9.4	446	2.09	1.81	9.9
	10	460	2.10	1.58	9.8	403	2.10	1.80	9.9
	100	483	2.08	1.60	10	438	2.09	2.06	9.8
	1000	476	2.09	1.73	9.7	457	2.09	1.57	10
PC-3	0	301	2.11	0.93	10	290	2.13	1.51	9.8
	1	288	2.10	1.42	10	349	2.08	1.53	9.7
	10	364	2.09	2.09	9.9	364	2.09	1.88	9.7
	100	333	2.10	2.10	9.7	323	2.10	1.34	9.6
	1000	266	2.08	2.08	9.6	301	2.10	1.68	9.5

Table S3. Ranking of candidate reference genes calculated by NormFinder based on their expression stability values.

Rank	0 nM		1 nM		10 nM		100 nM		1000 nM		Total samples	
	gene	SV	gene	SV	gene	SV	gene	SV	gene	SV	gene	SV
1	*GAPDH*	0.13	*ACTB*	0.06	*GAPDH*	0.06	*ACTB*	0.07	*ACTB*	0.03	*ACTB*	0.08
2	*ACTB*	0.14	*GAPDH*	0.13	*ACTB*	0.09	*GAPDH*	0.11	*GAPDH*	0.09	*GAPDH*	0.09
3	*TBP*	0.15	*TBP*	0.14	*TBP*	0.21	*18S rRNA*	0.17	*TBP*	0.24	*TBP*	0.18
4	*HPRT1*	0.25	*HPRT1*	0.25	*RPL13A*	0.27	*K-ALPHA-1*	0.21	*ALAS1*	0.29	*HPRT1*	0.26
5	*SDHA*	0.29	*SDHA*	0.27	*SDHA*	0.27	*TBP*	0.23	*HPRT1*	0.29	*SDHA*	0.27
6	*RPL13A*	0.34	*RPL13A*	0.30	*HPRT1*	0.28	*RPL13A*	0.28	*YWHAZ*	0.30	*RPL13A*	0.27
7	*K-ALPHA-1*	0.35	*K-ALPHA-1*	0.32	*K-ALPHA-1*	0.29	*SDHA*	0.29	*RPL13A*	0.31	*K-ALPHA-1*	0.28
8	*18S rRNA*	0.35	*YWHAZ*	0.32	*YWHAZ*	0.31	*YWHAZ*	0.29	*K-ALPHA-1*	0.32	*18S rRNA*	0.29
9	*ALAS1*	0.38	*ALAS1*	0.32	*18S rRNA*	0.32	*HPRT1*	0.33	*18S rRNA*	0.33	*YWHAZ*	0.30
10	*YWHAZ*	0.39	*18S rRNA*	0.38	*ALAS1*	0.33	*ALAS1*	0.33	*SDHA*	0.33	*ALAS1*	0.30
1	*miR-1228-3p*	0.20	*miR-16*	0.14	*miR-1228-3p*	0.10	*miR-1225-3p*	0.12	*miR-1225-3p*	0.11	*miR-1225-3p*	0.13
2	*miR-1225-3p*	0.22	*miR-1225-3p*	0.22	*miR-1225-3p*	0.12	*miR-16*	0.13	*miR-16*	0.20	*miR-16*	0.26
3	*RNU6-2*	0.28	*miR-130b*	0.33	*RNU6-2*	0.27	*miR-130b*	0.13	*miR-130b*	0.38	*miR-1228-3p*	0.31
4	*miR-16*	0.31	*RNU43*	0.36	*miR-130b*	0.42	*miR-1228-3p*	0.44	*RNU43*	0.43	*miR-130b*	0.35
5	*RNU43*	0.40	*miR-1228-3p*	0.37	*miR-16*	0.44	*RNU43*	0.51	*miR-1228-3p*	0.46	*RNU43*	0.41
6	*miR-130b*	0.42	*RNU6-2*	0.55	*RNU43*	0.45	*RNU6-2*	0.59	*RNU6-2*	0.61	*RNU6-2*	0.44

Table S4. Ranking of candidate reference genes calculated by BestKeeper based on their standard deviation.

Rank	0 nM		1 nM		10 nM		100 nM		1000 nM		Total samples	
	**gene**	**SD**	**gene**	**SD**	**gene**	**SD**	**gene**	**SD**	**gene**	**SD**	**gene**	**SD**
1	*ACTB*	0.23	*ACTB*	0.16	*ACTB*	0.17	*ACTB*	0.12	*ACTB*	0.15	*ACTB*	0.17
2	*GAPDH*	0.25	*GAPDH*	0.22	*YWHAZ*	0.19	*18S rRNA*	0.16	*YWHAZ*	0.20	*YWHAZ*	0.23
3	*TBP*	0.29	*YWHAZ*	0.23	*RPL13A*	0.19	*GAPDH*	0.21	*RPL13A*	0.22	*RPL13A*	0.24
4	*RPL13A*	0.30	*RPL13A*	0.25	*GAPDH*	0.24	*YWHAZ*	0.22	*ALAS1*	0.25	*GAPDH*	0.25
5	*YWHAZ*	0.32	*TBP*	0.26	*ALAS1*	0.32	*RPL13A*	0.25	*18S rRNA*	0.29	*18S rRNA*	0.30
6	*18S rRNA*	0.33	*ALAS1*	0.31	*18S rRNA*	0.34	*K-ALPHA-1*	0.30	*GAPDH*	0.31	*ALAS1*	0.31
7	*ALAS1*	0.34	*SDHA*	0.32	*SDHA*	0.34	*SDHA*	0.32	*TBP*	0.39	*TBP*	0.33
8	*SDHA*	0.36	*18S rRNA*	0.39	*TBP*	0.38	*ALAS1*	0.32	*SDHA*	0.43	*SDHA*	0.35
9	*HPRT1*	0.39	*K-ALPHA-1*	0.43	*K-ALPHA-1*	0.45	*TBP*	0.33	*K-ALPHA-1*	0.47	*K-ALPHA-1*	0.42
10	*K-ALPHA-1*	0.44	*HPRT1*	0.45	*HPRT1*	0.49	*HPRT1*	0.48	*HPRT1*	0.48	*HPRT1*	0.46
1	*miR-16*	0.31	*miR-16*	0.19	*miR-1228-3p*	0.09	*miR-1225-3p*	0.22	*miR-16*	0.19	*miR-16*	0.29
2	*miR-1228-3p*	0.36	*miR-1225-3p*	0.28	*RNU43*	0.29	*miR-16*	0.33	*miR-1225-3p*	0.25	*miR-1228-3p*	0.32
3	*RNU43*	0.42	*miR-1228-3p*	0.32	*miR-1225-3p*	0.30	*miR-1228-3p*	0.38	*miR-1228-3p*	0.38	*miR-1225-3p*	0.32
4	*miR-130b*	0.45	*miR-130b*	0.43	*miR-16*	0.40	*miR-130b*	0.41	*miR-130b*	0.44	*RNU43*	0.53
5	*miR-1225-3p*	0.47	*RNU43*	0.45	*RNU6-2*	0.56	*RNU43*	0.59	*RNU6-2*	0.63	*miR-130b*	0.54
6	*RNU6-2*	0.50	*RNU6-2*	0.56	*miR-130b*	0.68	*RNU6-2*	0.82	*RNU43*	0.64	*RNU6-2*	0.63

Table S5. Information on primers for amplification mRNA reference genes and target gene. *Not available.

Gene	Forward and Reverse Primer	Primer concentration (nM)	Product (bp)	Intron spanning	Reference
18S rRNA	GGATGTAAAGGATGGAAAATACA	50	72	NA*	[1]
	TCCAGGTCTTCACGGAGCTTGTT	50			
ACTB	TGACGTGGACATCCGCAAAG	50	205	Yes	[1]
	CTGGAAGGTGGACAGCGAGG	50			
ALAS1	AGTGTGAAAACCGATGGAGG	200	140	Yes	[2]
	CGATCATACTGAAAAGTGGAAACAG	200			
GAPDH	GACAGTCAGCCGCATCTTCT	50	127	Yes	[1]
	TTAAAAGCAGCCCTGGTGAC	50			
HPRT1	GACCAGTCAACAGGGGACAT	50	132	Yes	[3]
	CCTGACCAAGGAAAGCAAAG	50			
K-ALPHA-1	TGGAACCCACAGTCATTGATGA	200	135	Yes	[4]
	TGATCTCCTTGCCAATGGTGTA	200			
RPL13A	CGGACCGTGCGAGGTAT	200	114	Yes	[4]
	CACCATCCGCTTTTTCTTGTC	200			
SDHA	TGGTTGTCTTTGGTCGGG	50	85	Yes	[5]
	GCGTTTGGTTTAATTGGAGGG	50			
TBP	GAGAGTTCTGGGATTGTACCG	200	143	Yes	[5]
	ATCCTCATGATTACCGCAGC	200			
YWHAZ	ATGCAACCAACACATCCTATC	50	178	Yes	[6]
	GCATTATTAGCGTGCTGTCTT	50			

18S rRNA

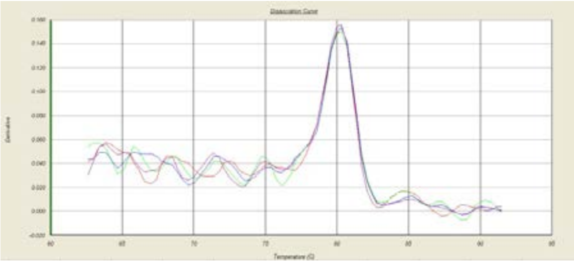

ACTB

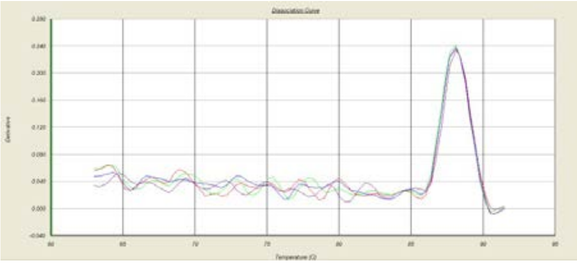

ALAS1

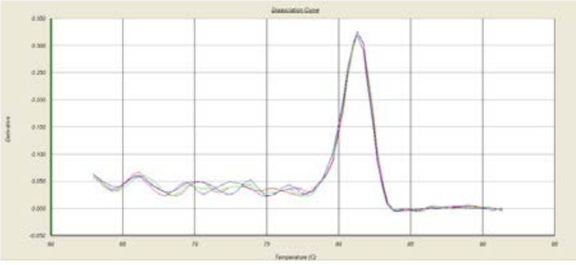

GAPDH

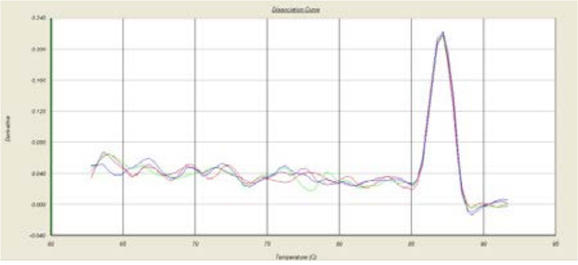

HPRT1

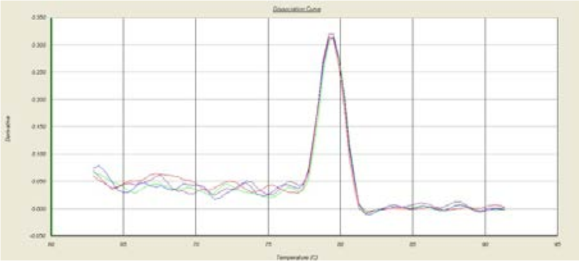

K-ALPHA-1

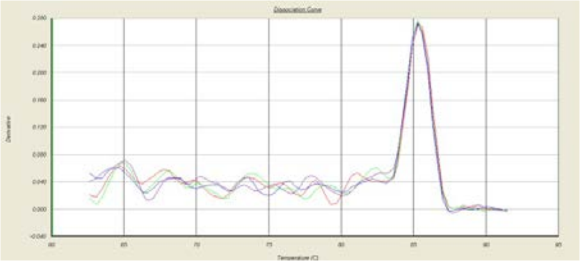

RPL13A

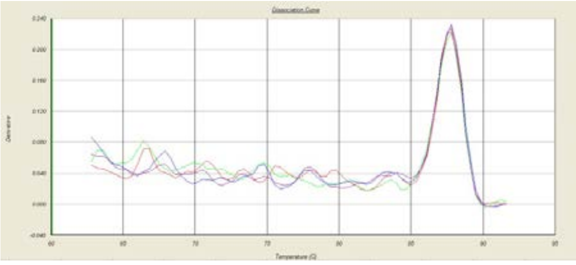

SDHA

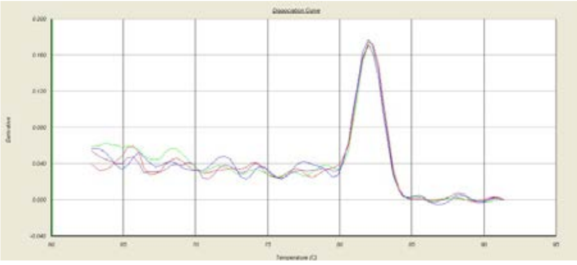

TBP

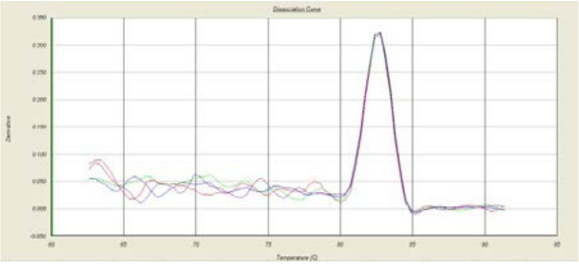

YWHAZ

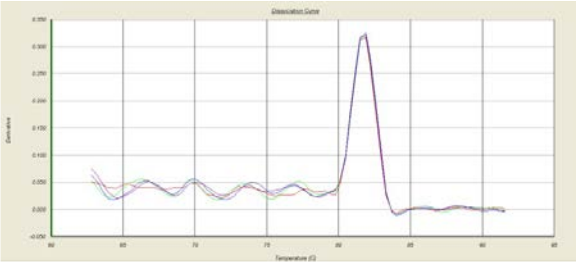

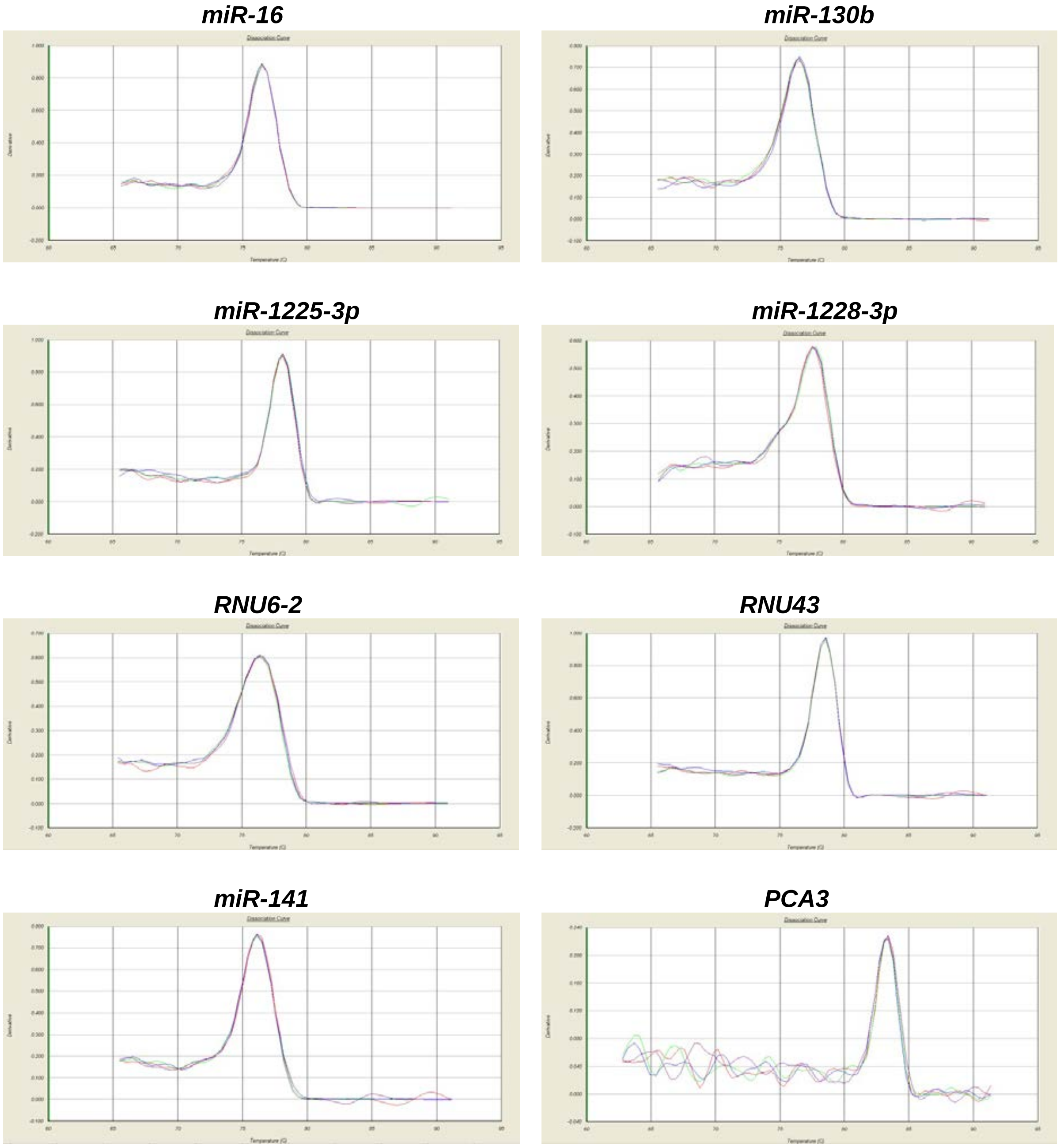
miR-16
miR-130b
miR-1225-3p
miR-1228-3p
RNU6-2
RNU43
miR-141
PCA3

Fig. S1

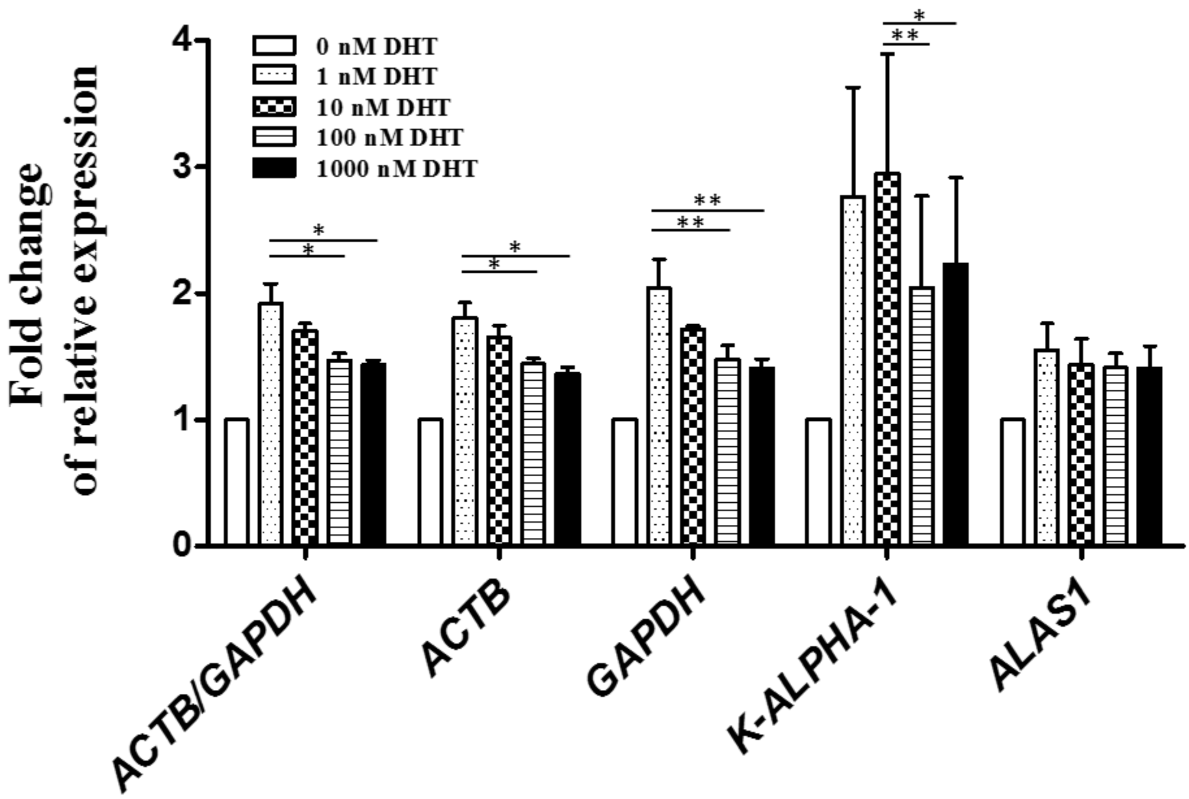
Fold change of relative expression
0 nM DHT
1 nM DHT
10 nM DHT
100 nM DHT
1000 nM DHT
0
1
2
3
4
*
**
ACTB/GAPDH
ACTB
GAPDH
K-ALPHA-1
ALAS1

Fig. S2

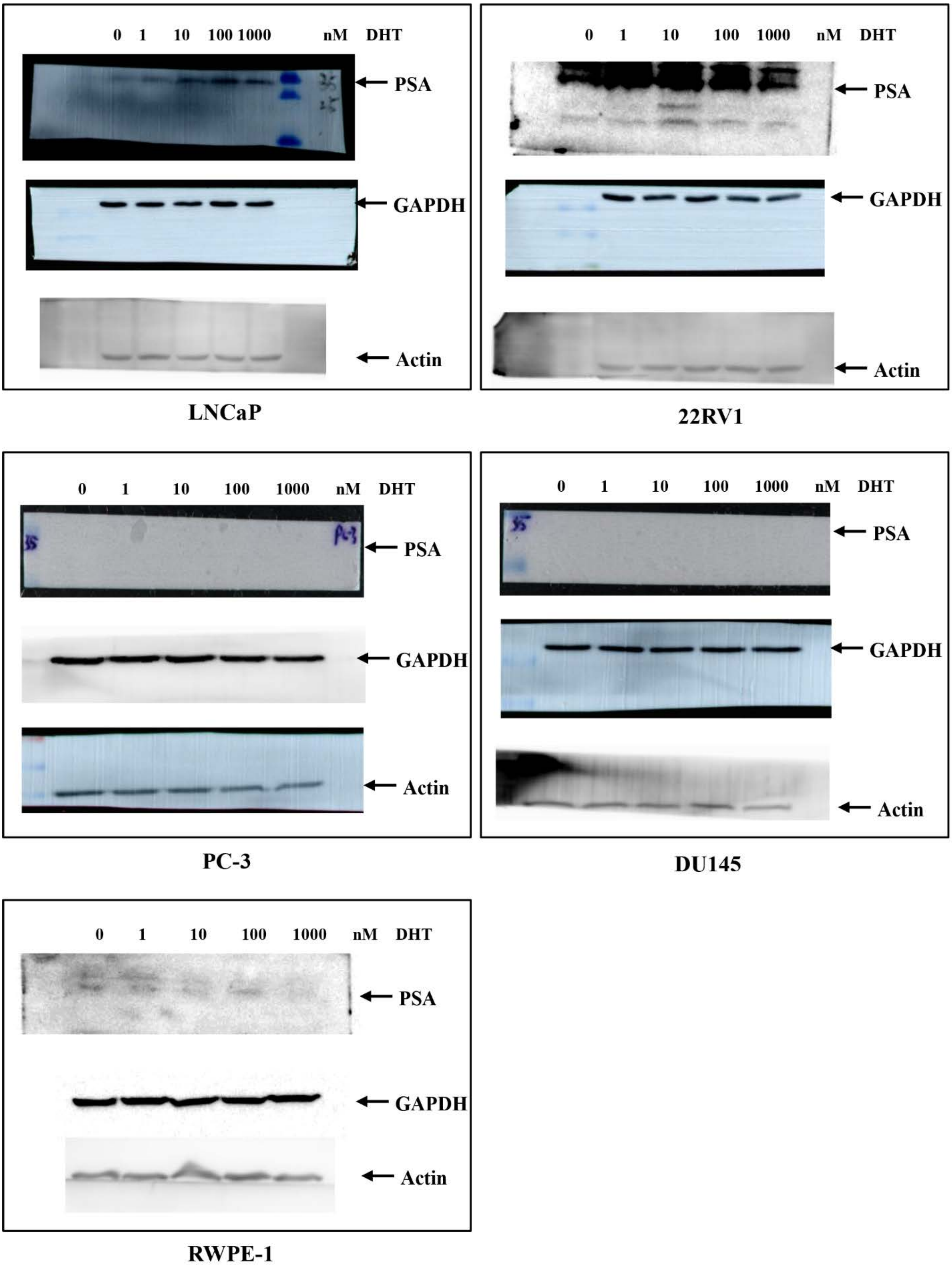
0 1 10 100 1000 nM DHT
PSA
GAPDH
Actin
LNCaP
0 1 10 100 1000 nM DHT
PSA
GAPDH
Actin
22RV1
0 1 10 100 1000 nM DHT
PSA
GAPDH
Actin
PC-3
0 1 10 100 1000 nM DHT
PSA
GAPDH
Actin
DU145
0 1 10 100 1000 nM DHT
PSA
GAPDH
Actin
RWPE-1

Fig. S3

Supplementary References

1. Liu, L. L. *et al.* Identification of valid reference genes for the normalization of RT-qPCR expression studies in human breast cancer cell lines treated with and without transient transfection. *PLoS One.* **10,** e0117058 (2015).
2. Souza, A. F., Brum, I. S., Neto, B.S., Berger, M. &Branchini, G. Reference gene for primary culture of prostate cancer cells. *Mol. Biol. Rep.* **40(4),** 2955-2962 (2013).
3. Li, Y. L., Ye, F., Hu, Y., Lu, W. G. & Xie, X. Identification of suitable reference genes for gene expression studies of human serous ovarian cancer by real-time polymerase chain reaction. *Anal. Biochem.* **394(1),** 110-116 (2009).
4. Ohl, F. *et al.* Gene expression studies in prostate cancer tissue: which reference gene should be selected for normalization? *J. Mol. Med. (Berl).* **83(12),** 1014-1024 (2005).
5. Ceelen, L. *et al.* Critical selection of reliable reference genes for gene expression study in the HepaRG cell line. *Biochem. Pharmacol.* **81(10),** 1255-1261(2011).
6. De Spiegelaere, W. *et al.* Elimination of amplification artifacts in real-time reverse transcription PCR using laser capture microdissected samples. *Anal. Biochem.* **382(1),** 72-74 (2008).
